# GIP in Cardiovascular and Kidney Disease: From Physiology to Pharmacology

**DOI:** 10.1111/dom.70888

**Published:** 2026-05-28

**Authors:** Michelantonio De Fano, Line L. Haurum, Christine R. Schwarz, Francesca Porcellati, Andreas Andersen

**Affiliations:** ^1^ Department of Medicine and Surgery Perugia University Medical School Perugia Italy; ^2^ Clinical and Translational Research, Copenhagen University Hospital – Steno Diabetes Center Copenhagen Herlev Denmark

**Keywords:** cardiovascular disease, GIP, incretin therapy, obesity therapy, type 2 diabetes

## Abstract

**Aims:**

To provide a comprehensive overview of the cardiovascular and renal effects of glucose‐dependent insulinotropic polypeptide (GIP) by integrating its physiological role with recent human trial data on tirzepatide, the first dual GIP and glucagon‐like peptide‐1 (GLP‐1) receptor agonist.

**Materials and Methods:**

This narrative review synthesizes key physiological data of native GIP across tissues—especially the heart, vessels, kidney, and adipose tissue—and summarizes clinical evidence from the SUMMIT and the SURPASS programmes. Central to this review is the analysis of the SURPASS‐CVOT trial, which compared the cardiovascular protective effects of tirzepatide versus dulaglutide in individuals with type 2 diabetes and established atherosclerotic CV disease.

**Results:**

Preclinical data regarding GIP receptor modulation in the cardiorenal district remains controversial, a contention partially reflected in recent clinical evidence. Within the innovative framework of SURPASS‐CVOT, tirzepatide established non‐inferiority to dulaglutide for 3‐point MACE while slowing eGFR decline in participants with high‐risk chronic kidney disease. Furthermore, the SUMMIT trial demonstrated that in individuals with obesity and heart failure (HF) with preserved ejection fraction, tirzepatide reduced the composite endpoint of CV death or worsening HF events, regardless of baseline kidney function. Despite these advancements, knowledge gaps persist regarding the potential synergistic cardiorenal benefits of combining dual incretin agonism with sodium‐glucose cotransporter‐2 inhibitors.

**Conclusion:**

While adding GIP receptor agonism to GLP‐1 receptor agonism clearly improves cardiometabolic risk factors, its precise and independent contribution to long‐term CV and renal protection remains to be fully elucidated. Ongoing outcome trials, such as SURMOUNT‐MMO and TREASURE‐CKD, will provide additional insights into these effects. Furthermore, emerging strategies—including combining GIP receptor antagonism with GLP‐1 receptor agonism or moving toward triple agonism—represent promising alternatives to favorably modify the multiple determinants of cardiometabolic risk.

## 1 Introduction

Glucagon‐like peptide‐1 (GLP‐1) receptor agonists (GLP‐1 RAs) have transformed the therapeutic management of type 2 diabetes (T2D) and are revolutionizing obesity treatment. Beyond efficacy in glycaemic control and weight reduction, several agents within this class have demonstrated beneficial effects on cardiovascular (CV) disease and chronic kidney disease (CKD), which are among the most prevalent complications associated with T2D and obesity [1, 2].

In T2D, a recent meta‐analysis of CV outcomes trials (CVOTs) revealed that GLP‐1 RAs reduce major adverse CV events (MACE) by 14%, hospitalization for heart failure (HF) by 14% and a composite kidney outcome by 17% [3]. Specifically, albiglutide, liraglutide, dulaglutide, efpeglenatide and semaglutide (injectable and oral) have demonstrated significant CV benefits [4, 5, 6, 7, 8, 9], whereas injectable semaglutide also showed renal protection in a dedicated kidney outcome trial involving people with T2D and CKD [10].

In individuals with overweight or obesity and established CV disease without diabetes, once‐weekly (QW) subcutaneous (SC) semaglutide 2.4 mg reduced MACE in the SELECT trial [11]. Dedicated kidney outcome trials in individuals with obesity and CKD are still lacking; however, prespecified kidney analyses from SELECT suggest kidney benefits of semaglutide [12].

The emerging frontier in incretin‐based therapy involves targeting multiple receptors. The first dual incretin agonist approved for the treatment of T2D and obesity is tirzepatide, a combined GLP‐1R and glucose‐dependent insulinotropic polypeptide (GIP) receptor (GIPR) agonist. Its efficacy and safety were assessed in the SURPASS programme for T2D and the SURMOUNT programme for obesity [13]. Tirzepatide has also been investigated in individuals with HF with preserved ejection fraction (HFpEF) in the SUMMIT trial [14]. Notably, more recently, SURPASS‐CVOT compared tirzepatide with dulaglutide in individuals with T2D and established CV disease [15].

The scope of this review is to outline the physiology of GIP, with particular emphasis on cardiovascular and renal biology, and to discuss how current pharmacological strategies—particularly dual incretin agonism—inform the evolving therapeutic role of GIP in cardiorenal disease.

## 2 Historical Background

The relationship between the gastrointestinal (GI) tract and glucose metabolism emerged in the early 20th century, when duodenal extracts were shown to reduce glycosuria in individuals with diabetes, suggesting that gut‐derived factors modulate pancreatic endocrine function. A major conceptual advance came with the recognition that oral glucose elicits a greater insulin response than intravenous glucose, a phenomenon later termed *the incretin effect* [16]. GIP was subsequently isolated from intestinal mucosa and initially described as an inhibitor of gastric acid secretion, leading to term *gastric inhibitory polypeptide* [17]. Its predominant physiological role was later identified as glucose‐dependent stimulation of insulin secretion, prompting its renaming as *glucose‐dependent insulinotropic polypeptide* [18].

Molecular studies in the early 1980s identified GLP‐1 (7–36) amide as a second incretin hormone with potent insulinotropic activity [19]. Experimental models showed that antagonism or deletion of both GIPR and GLP‐1R markedly reduces the incretin effect, confirming their complementary roles [20].

Notably, the incretin effect is markedly reduced in T2D. GIP's insulinotropic response becomes profoundly attenuated, even at supraphysiological concentrations, and preclinical studies linked GIP signalling to weight gain [21, 22]. Conversely, GLP‐1 largely preserves its insulinotropic properties [23, 24, 25]. Consequently, GIP received less therapeutic attention than GLP‐1 for decades [26].

## 3 Physiology of Native GIP

### 3.1 Secretion

GIP is a 42‐amino acid peptide hormone secreted by enteroendocrine K cells, predominantly located in the duodenum and proximal jejunum, with lower density distally [27]. Like other peptides of the secretin/vasoactive intestinal polypeptide (VIP) family, active GIP is synthesized from prepro‐GIP through post‐translational cleavage [22].

Fasting GIP plasma concentrations are low (picomolar) [28], rising rapidly after nutrient ingestion and peaking 30–60 min after oral glucose or mixed meals [22]. Carbohydrates and dietary fat strongly stimulate GIP secretion, while recent evidence shows protein can also elicit robust responses, in some cases rising faster than after fat consumption [29]. Postprandial GIP typically exceeds GLP‐1 concentrations [30], with considerable inter‐individual variability [31].

Like GLP‐1, GIP is rapidly inactivated by the protease dipeptidyl peptidase‐4 (DPP‐4), with additional peptides and renal clearance contributing to its degradation, resulting in a half‐life of ~5–7 min [32]. Some circulating GIP‐related fragments may also exert noncanonical effects independent of GIPR signalling [33].

**FIGURE 1:**
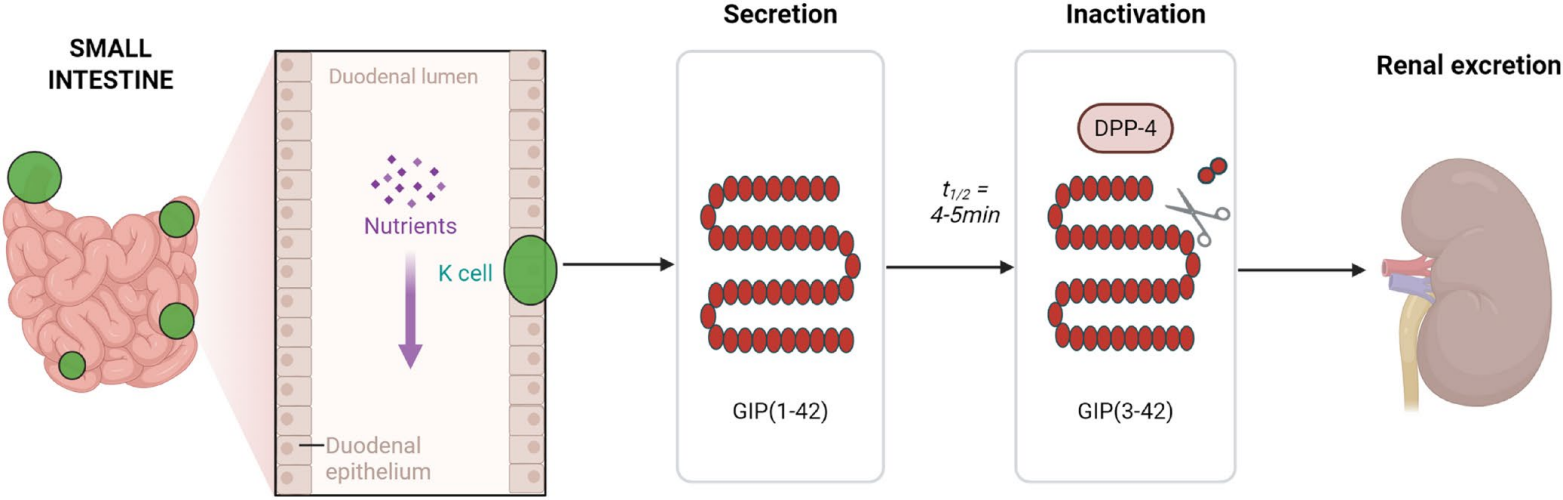
Summary of GIP physiology. The green dots progressively decrease in size along the small intestine, reflecting a decreasing density of GIP secretion. In the intestinal detail, the green dot indicates the localization of K cells. Abbreviations: DPP‐4: dipeptidyl peptidase‐4, GIP: glucose‐dependent insulinotropic polypeptide.

Figure 1 schematizes the physiology of GIP (Figure 1).

### 3.2 GIP Receptor

GIP binds the GIPR, a class B G‐protein–coupled receptor sharing ~40% sequence homology with GLP‐1R and 44% with the glucagon receptor (GcgR) [34]. Genetic variants, including a carboxy‐terminally truncated isoform, can reduce receptor activity when co‐expressed with full‐length GIPR in β‐cells [35].

## 4 Actions of GIP Across Tissues

### 4.1 Pancreas

GIPR is abundantly expressed on β‐cells, but also on α‐cells (possibly *δ* and PP‐cells), whereas GLP‐1R is more β‐cell–enriched [27]. GIP enhances insulin secretion in a glucose‐dependent manner [18], accounting for 60%–80% of postprandial insulin secretion in healthy individuals [21, 22, 36] and stimulates glucagon release primarily during fasting or hypoglycaemia.

Hyperglycaemic clamp studies demonstrated that GIP's insulinotropic effect is markedly reduced in T2D and type 1 diabetes (T1D) [37], a phenomenon termed “GIP resistance” [38], whereas GLP‐1 activity is only partially impaired [39]. This impairment may result from GIPR desensitization induced by elevated GIP concentrations, causing receptor downregulation via slowed recycling [40, 41].

Experimental studies further suggest pro‐survival actions of GIP signalling in β‐cells, although their translational relevance in humans remains uncertain [42, 43].

### 4.2 Heart and Blood Vessels

GIPR is expressed in CV tissues, including the heart and vasculature, although its precise physiological and pathophysiological roles remain incompletely elucidated and appear, in some respects, paradoxical [44].

In humans, infusion studies suggest that GIP exerts direct CV effects independent of its insulinotropic action. Heart rate increases during GIP infusion in healthy individuals and in those with obesity, T1D and T2D [45, 46, 47], possibly reflecting cholinergic stimulation [45]. In parallel, GIP infusion appears to reduce diastolic BP in T1D [48, 49] and lower mean arterial pressure in healthy individuals as well as in those with impaired glucose tolerance and T2D [46, 50]. The clinical relevance of these acute hemodynamic responses, particularly in the context of long‐term GIP‐based therapies, remains uncertain.

Preclinical studies suggest several potential cardioprotective actions of GIP signalling. In animal models, GIP reduces myocardial triglyceride (TG) content by increasing phosphorylated hormone‐sensitive lipase and fatty acid (FA) oxidation [51]. It also downregulates pro‐hypertrophic and pro‐fibrotic genes, including TGF‐β, MMP9, COL1A1, MuRF1 and FBXO32, suggesting favourable effects on cardiac structure and metabolism under metabolic stress [52]. Furthermore, cardiomyocyte GIPR activation increases SERCA2 expression and phospholamban phosphorylation while limiting hyperactivation of PKA and CaMKII, thereby enhancing sarcoplasmatic reticulum calcium reuptake and excitation‐contraction coupling [52].

Within the vascular system, GIP has been shown to increase postprandial splanchnic blood flow to the pancreas and jejunum, although not to the duodenum, suggesting a role in regional hemodynamic adaptation to nutrient absorption [53].

In vitro and animal studies indicate that GIP may influence atherosclerosis‐related pathways, although findings remain heterogeneous. Reported anti‐atherogenic effects include increased nitric oxide production, reduced foam cell formation and inhibition of vascular smooth muscle cell (VSMC) proliferation. In atherosclerosis‐prone ApoE^−^/^−^ mice, GIP overexpression reduced macrophage infiltration and increased collagen within plaques, enhancing stability without altering lesion size [54, 55]. These findings support a potential anti‐inflammatory and plaque‐stabilizing role of GIP in experimental atherosclerosis.

Conversely, pro‐atherogenic effects have been described in vitro, including increased endothelin‐1 and osteopontin (OPN) production in endothelial cells and VSMCs, respectively [56]. The relative contribution of these opposing mechanisms in vivo remains unclear.

In observational studies in humans, circulating GIP levels have been reported to be elevated in individuals with peripheral artery disease, although not directly associated with coronary artery disease per se [54], suggesting a link between GIP signalling and peripheral atherosclerotic burden.

At the molecular level, GIPR polymorphisms and circulating GIP levels have been associated with elevated OPN levels, a protein implicated in vascular inflammation, plaque progression and adverse CV outcomes [57]. Moreover, individuals with prior major CV events exhibit increased circulating levels of OPN and GIP [56]. Whether these associations reflect compensatory protective responses or contribute directly to disease pathogenesis remains unresolved.

### 4.3 Kidney

Unlike GLP‐1R, GIPR expression appears low or absent in human kidney tissue, and its precise localization within renal structures remains incompletely characterized [42].

In experimental studies in rats, supraphysiological doses of GIP induced vasoconstriction in splanchnic organs, including the kidney, through mechanisms that remained undefined [58]. These findings suggest that GIP may influence renal vascular tone under specific experimental conditions; however, relevance at physiological or therapeutic concentrations remains uncertain, and the translational significance of these preclinical vascular observations is unclear. To date, no consistent evidence shows clinically meaningful changes in renal perfusion or function attributable to GIP signalling in humans.

Direct evidence linking GIP signalling to kidney protection in humans therefore remains limited. Potential relevance is more likely to involve indirect mechanisms, including improved systemic metabolic control, insulin sensitivity, lipid handling and potential anti‐atherogenic effects benefiting renal microvascular health. Whether these pathways translate into clinically meaningful renal protection with GIP‐based therapies remains to be determined.

Particular interest has focused on perirenal and intrarenal adipose tissue (AT), which are increasingly viewed as active mediators of renal inflammation in metabolic disease and where the presence of GIPR is well‐documented [59]. Experimental data suggest inflammatory signalling from these depots can contribute to local oxidative stress, immune cell infiltration and progression of kidney damage [60, 61, 62]. GIP signalling may therefore influence renal outcomes indirectly through modulation of AT inflammation and metabolic function.

**FIGURE 2:**
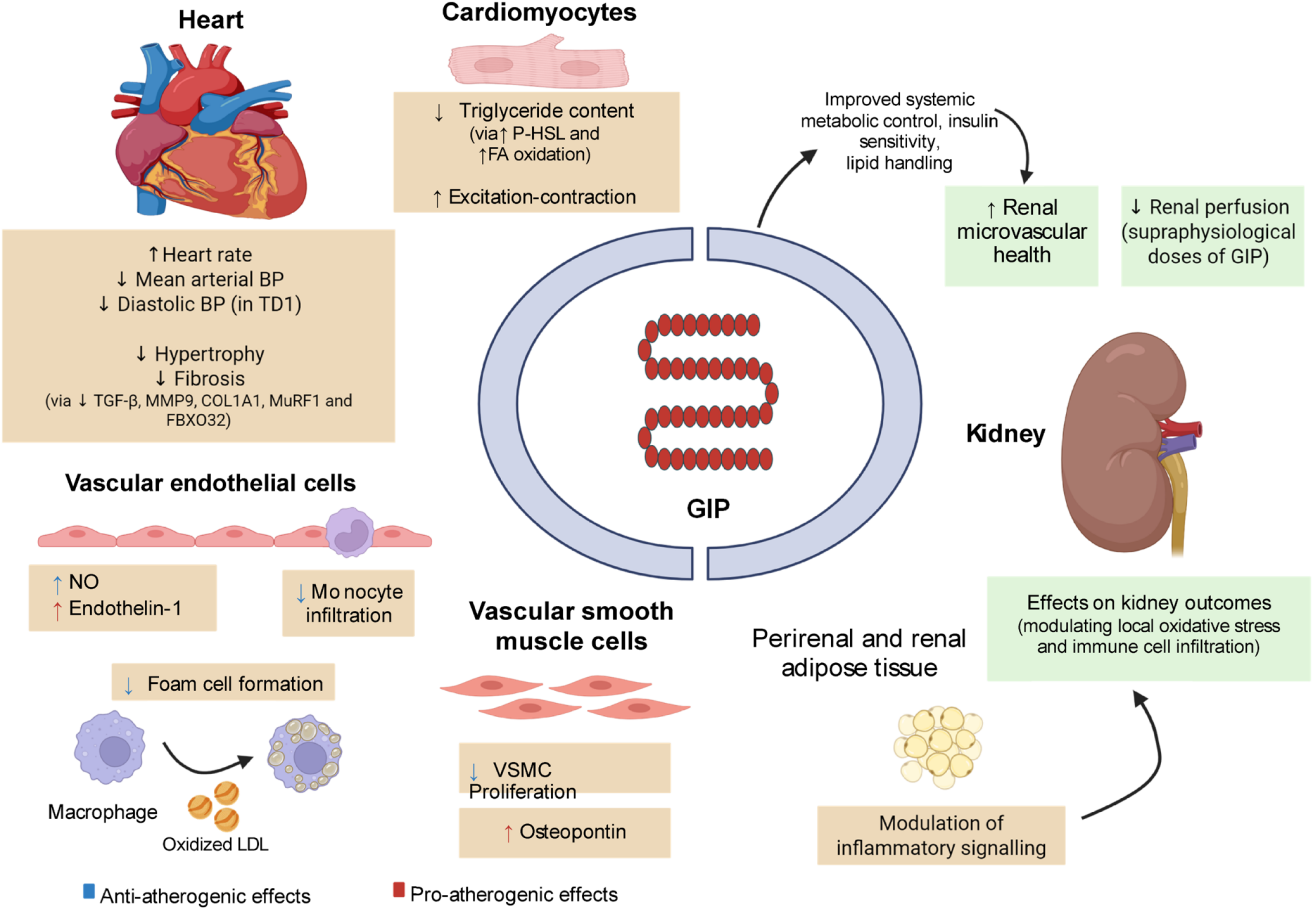
Cardiovascular and renal effects of GIPR agonism. Brown boxes indicate effects resulting from direct GIP‐GIPR binding, while the green box represents effects mediated by indirect signalling. Effects on the heart: Observed in both preclinical (reduction in hypertrophy, fibrosis, myocardial lipid accumulation and increase in coupling excitation‐contraction) and clinical studies (increased in heart rate). Effects on arteries and kidney: All observed in preclinical models. Abbreviations: BP: blood pressure, COL1A1: collagen Type I Alpha 1 Chain, FA: fatty acid, FBXO32: F‐box protein 32, GIP: glucose‐dependent insulinotropic polypeptide, LDL: low‐density lipoprotein, MMP9: matrix metalloproteinase‐9, MuRF1: muscle RING‐finger protein‐1, NO: nitric oxide, P‐HSL: phosphorylated hormone‐sensitive lipase, TD1: type 1 diabetes, TGF‐β: transforming growth factor‐beta, VSMC: vascular smooth muscle cells.

Figure 2 illustrates the main effects of GIP agonism in the CV system and kidney.

### 4.4 Adipose Tissue

Against this background, AT warrants closer consideration, since the indirect CV and renal implications outlined above coexist with highly context‐dependent local effects of GIP signalling.

White adipose tissue (WAT) expresses GIPR [42], where GIP enhances lipoprotein lipase (LPL) activity, a key determinant of postprandial TGs clearance. Through LPL‐mediated hydrolysis, FAs become available for lipogenesis, supporting the concept that GIP facilitates nutrients storage during energy surplus [63].

This interpretation is supported by in vivo data from Murphy et al., showing parallel postprandial increases in plasma GIP and LPL after lipid‐rich meals [64], both declining toward fasting levels as fat storage demand decreases. Under hyperinsulinemic‐hyperglycaemic clamp conditions, mimicking the post‐prandial state, GIP further increases WAT blood flow and amplifies insulin‐induced TGs hydrolysis [65], an effect maintained even when endogenous insulin secretion is suppressed [66], suggesting partial insulin‐independent actions.

GIP also promotes adipocyte glucose uptake, both directly via insulin‐mimetic lipogenic effects and indirectly through enhanced insulin secretion, thereby favouring FA synthesis from glucose [67].

The functional redundancy between GIP and insulin may represent an evolutionary mechanism to preserve energy during nutrient scarcity. In line with this, clinical stimulation studies showed higher BMI predicts greater circulating GIP responses [68]. However, this metabolic profile is not uniformly beneficial: in vitro studies in human adipocytes indicate that GIP can impair insulin‐stimulated glucose uptake under certain conditions, suggesting that its effects may shift toward insulin resistance depending on metabolic context [69].

GIPR is also expressed in brown adipose tissue (BAT), where murine studies indicate it upregulates thermogenic and catabolic genes [70], although whether these findings translate into meaningful effects in humans remains uncertain.

An additional level of complexity, already suggested by the inflammatory mechanisms discussed in the renal context, concerns the interaction between GIP signalling and immune pathways within AT. GIPR is present on monocytes, macrophages and selected bone marrow T cells, indicating that GIP signalling is active. In animal models, GIPR activation reduces pro‐inflammatory cytokine expression and macrophage infiltration, albeit predominantly in conjunction with weight loss [71]. Conversely, acute GIP infusion in individuals with obesity increases chemokines CCL2, CCL8 and IL‐6 [72]. Consistently, in a cohort of 168 individuals with obesity, higher plasma GIP levels were associated with upregulation of 32 inflammation‐related genes, greater visceral adiposity and increased circulating levels of IL‐6, CCL2 and VCAM‐1 [73], reinforcing the view that GIP‐related inflammatory effects may depend on the underlying metabolic milieu.

### 4.5 Other Tissues

Neither the liver nor skeletal muscle express incretin receptors; therefore, GIP and GLP‐1 act via indirect pathways [33]. In animal models, GIP enhances skeletal muscle glucose uptake and GLUT4 expression [74], reflecting enhanced insulin sensitivity. In individuals with T1D, short‐term GIP infusion increased hepatic lipid accumulation without altering circulating lipid species [75].

Within the central nervous system (CNS), GIPR is widely distributed (cerebral cortex, hypothalamus, pituitary, hippocampus, olfactory bulb, substantia nigra, cerebellum) though its ability to cross the blood–brain barrier remains uncertain [33]. Animal studies showed GIP analogues support progenitor cell proliferation, learning, memory and synaptic plasticity, and exert neuroprotective effects in models of Alzheimer's, Parkinson's and Huntington's diseases [76, 77].

GIP's role in appetite is complex: acute activation of GIPR neurons can reduce food intake, whereas long‐term treatment generally shows no effects [78]. However, long‐acting GIP agonists and GIP–GLP‐1 co‐agonists reduce body weight and food intake via inhibitory GABAergic neurons [79].

In the GI tract, GIP modulates GLP‐1 release and glucose transport independently of insulinotropic properties. Unlike GLP‐1, it minimally affects gastric motility but acts as an “enterogastrone”, inhibiting gastric acid secretion [80] and modestly contributing to postprandial nutrient handling.

GIPR expression in bone suggests a role in skeletal metabolism. Experimental studies demonstrate GIP promotes bone formation and inhibits resorption, partially independent of insulin and glycaemic control. In humans, acute GIP administration suppressed markers of bone resorption [49, 81], though the long‐term effects remain unknown.

**FIGURE 3:**
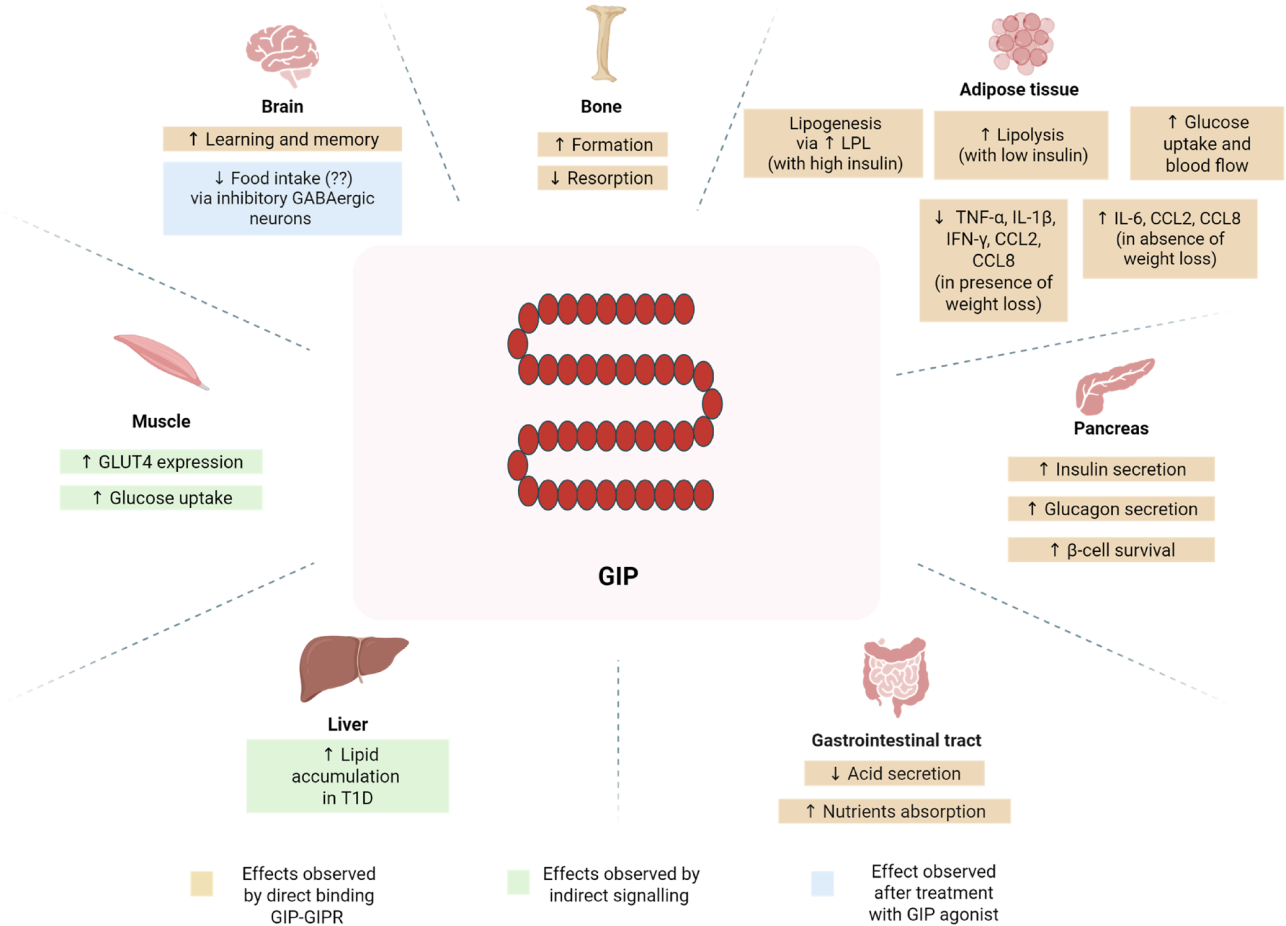
Effect of GIPR agonism across different tissues. Brown boxes indicate effects induced by direct GIP‐GIPR binding, green boxes denote effects arising from indirect signalling, and the light blue box indicates effect observed following treatment with GIP agonist. Skeletal muscle: Effects observed in preclinical models. Pancreas: Effects demonstrated in both preclinical (increased β‐cell survival) and clinical studies (increased insulin and glucagon secretion). Liver: Effects observed in clinical studies. Bone: Effects demonstrated in both preclinical and clinical studies. Adipose tissue: Effects observed in preclinical studies (increased lipolysis and lipogenesis depending on insulin presence; increased or reduced inflammation depending on weight change; increased glucose uptake) and in clinical studies (increased lipolysis and adipose tissue blood flow). Gastrointestinal tract: Effects demonstrated in preclinical models. Brain: Effects demonstrated in preclinical models. Abbreviations: CCL2: CC motif chemokine ligand 2, CCL8: CC motif chemokine ligand 8, GABA: gamma‐aminobutyric acid, GIP: glucose‐dependent insulinotropic polypeptide, GLUT4: glucose transporter type 4, IFN‐γ: interferon‐gamma, IL‐1β: interleukin‐1 beta, IL‐6: Interleukin‐6, LPL: lipoprotein lipase, T1D: type 1 diabetes, TNF‐α: tumour necrosis factor‐alpha.

Figure 3 summarizes the effects of GIP agonism in other tissues (Figure 3).

## 5 GIPR As Pharmacological Target

In accordance with the above pathophysiological considerations, early therapeutic development of GIPR agonists as pharmaceutical agents was limited. Concerns regarding the metabolic relevance of GIP signalling in obesity and T2D, including impaired insulinotropic effects in these conditions, contributed to a prolonged lack of interest in GIPR agonism for clinical use. Consequently, initial preclinical studies with stable GIPR agonists did not advance to clinical development until relatively recently [43].

A major conceptual shift occurred in 2013 with the description of unimolecular peptide co‐agonists exhibiting balanced affinity and efficacy at both the GIPR and GLP‐1R [82]. These agents were developed not only based on evidence that dual receptor activation could improve glucose metabolism, but also following the unexpected observation of greater weight loss and appetite suppression compared with GLP‐1R agonism alone in diet‐induced obese rodents [82, 83].

Early co‐agonists were engineered from the glucagon sequence, with stepwise incorporation of amino acid residues from GLP‐1, GIP and exendin‐4 to achieve near‐balanced dual agonism, while ensuring resistance to DPP‐4 degradation and eliminating activity at GcgR [82]. First‐generation DPP‐4‐protected co‐agonists, either pegylated or acylated with a C16 fatty acid, demonstrated superior metabolic efficacy compared with liraglutide across multiple animal models [82]. Among these compounds, the acylated dual agonist MAR709 reached clinical testing, but its development was discontinued following a 12‐week phase 2a trial due to only modest superiority over liraglutide in T2D [84]. Importantly, subsequent preclinical data confirmed a GIPR‐dependent mechanism underlying its enhanced weight‐reducing effects, providing critical proof‐of‐concept for GIPR engagement in the pharmacological field and supporting the shift toward dual incretin agonism in light of the limited efficacy of GIP mono‐agonism [79, 85].

## 6 Tirzepatide

Tirzepatide is a dual incretin co‐agonist that activates GLP‐1R and GIPR. This 39‐amino‐acid linear peptide is derived mainly from the human GIP sequence, with segments shared with native GLP‐1 and exendin‐4. Amino‐iso‐butyric acid substitutions at positions 2 and 13 confer resistance to DPP‐4 degradation, while an (AEEA)2‐γ‐Glu‐C20 diacid chain on lysine in position 20 enables albumin binding, extending the half‐life to ~5 days for once‐weekly SC administration [86].

Tirzepatide binds GIPR with native‐like affinity but has lower GLP‐1R potency, often described as an “imbalanced” dual agonist favouring GIPR signalling [87]. Moreover, tirzepatide exhibits GLP‐1R‐biased agonism, favouring cAMP signalling over β‐arrestin recruitment and reducing receptor internalization, which may enhance insulin secretion depending on experimental context [87].

Additionally, tirzepatide improves insulin sensitivity, lowers glucagon secretion, and—based on *vivo* models—reduces food intake and promotes greater weight loss than single agonists [88]. Phase 1 studies showed its pharmacokinetics are unaffected by renal or hepatic impairment [89].

## 7 Tirzepatide for the Treatment of Obesity

The SURMOUNT phase 3 program was designed to assess tirzepatide for obesity management, with particular relevance not only for weight reduction but also for the modification of cardiometabolic risk factors linked to CV morbidity and mortality [90]. Across published studies, the primary endpoint was percentage body weight change after 72 weeks (or study‐specific end‐of‐treatment), except in SURMOUNT‐OSA 1 and 2, where change in apnoea‐hypopnea index (AHI) at 52 weeks was the primary endpoint [91]. Key findings from the program are summarized in Table 1 [91, 92, 93, 94, 95, 96, 98].

**TABLE 1:** Overview of the SURMOUNT programme.

Trial	Participant characteristics	Duration	Outcomes	Results in treatment arms (expressed as treatment regimen estimand)			
SURMOUNT‐1 [92] (*n* = 2539)	Adults with obesity (BMI ≥ 30 kg/m^2^) or overweight (≥ 27 kg/m^2^) + at least one comorbidity^a^ Mean BMI 38 kg/m^2^	72 weeks	Primary outcome	Tirzepatide 5 mg	Tirzepatide 10 mg	Tirzepatide 15 mg	Placebo
			Superiority of TZP in body weight CFB (in %) vs. placebo (ETD vs. placebo)	−15.0 (−11.9) **	−19.5 (−16.4) **	−20.9 (−17.8) **	−3.1
			Outcomes on CV risk factors				
			Waist circumference (CFB in cm)	−14.0	−17.7	−18.5	−4.0
			Blood pressure sys/dia (CFB in mmHg)	−7.0/−5.2	−8.2/−5.5	−7.6/−4.6	−1.2/−1.0
			LDL (CFB in %)	−5.3	−6.6	−8.6	−0.9
SURMOUNT‐2 [93] (*n* = 938)	Adults with BMI ≥ 27 kg/m^2^ and T2D^b^ mean BMI 36.1 kg/m^2^	72 weeks	Primary outcomes	—	Tirzepatide 10 mg	Tirzepatide 15 mg	Placebo
			Superiority of TZP in body weight CFB (in %) vs. placebo (ETD vs. placebo)	—	−12.8 (−9.6) **	−14.7 (−11.6) **	−3.2
			Superiority of TZP in participants (in %) achieving ≥ 5% weight loss vs. placebo	—	79 OR 8.3 ** (95% CI 5.6, 12.3)	83 OR 10.5 ** (95% CI 6.8, 16.1)	32
			Outcomes on CV risk factors				
			Waist circumference (CFB in cm)	—	−10.8	−13.1	−3.3
			Blood pressure sys/dia (CFB in mmHg)	—	−6.3/−2.5 (pooled 10/15 mg)		−1.2
			LDL (CFB in %)	—	2.9 (pooled 10/15 mg)		7.4
SURMOUNT‐3 [94] (*n* = 806)	Adults with obesity (BMI ≥ 30 kg/m^2^) or overweight (≥ 27 kg/m^2^) + at least one comorbidity^a^ (who lost ≥ 5% following a 12‐week run‐in period with lifestyle intervention) mean BMI 38.6 kg/m^2^	72 weeks	Primary outcomes	—	Tirzepatide MTD (10 or 15 mg)		Placebo
			Superiority of TZP in body weight CFB (in %) vs. placebo (ETD vs. placebo)	—	−18.4 (−20.8) **		2.5
			Superiority of TZP in participants (in %) achieving ≥ 5% weight loss vs. placebo	—	87.5 OR 34.6 ** (95% CI 19.2, 62.6)		16.5
			Outcomes on CV risk factors				
			Waist circumference (CFB in cm)	—	−14.6		0.2
			Blood pressure sys/dia (CFB in mmHg)	—	−5.1/−3.2		4.1/2.3
			LDL (CFB in %)	—	−6.1		6.1
SURMOUNT‐4 [95] (*n* = 783)	Adults with obesity (BMI ≥ 30 kg/m^2^) or overweight (≥ 27 kg/m^2^) + at least one comorbidity^a^ mean BMI 38.4 kg/m^2^ Tirzepatide 10 or 15 mg versus placebo (following an open‐label period of tirzepatide treatment at the highest tolerated dose)	88 weeks (36 lead‐in +52 randomized)	Primary outcome	—	Continued tirzepatide (10 or 15 mg)		Placebo
			Superioriority of TZP in body weight CFB (in %) after withdrawal vs. continuation (ETD vs. placebo)	—	−5.5 (−19.4) **		14
			Outcomes on CV risk factors				
			Waist circumference (from week 36–88) in cm	—	−4.3		7.8
			Blood pressure sys/dia (change from week 36–88 in mmHg)^c^	—	2.1/−0.4		8.4/3.2
			LDL (CFB in %)	—	−5.2		2.6
SURMOUNT‐5 [96] (*n* = 751)	Adults with obesity (BMI ≥ 30 kg/m^2^) or overweight (≥ 27 kg/m^2^) + at least one comorbidity^a^ mean BMI 39.4 kg/m^2^	72 weeks	Primary outcome	—	Tirzepatide MTD (10 or 15 mg)		Semaglutide MTD (1.7 or 2.4 mg)
			Superiority of TZP in body weight CFB (in %) vs. semaglutide (ETD vs. semaglutide)	—	−20.2 (−6.5) **		−13.7
			Outcomes on CV risk factors				
			Waist circumference (CFB in cm)	—	−18.4		−13.0
			Blood pressure sys/dia (CFB in mmHg)^c^	—	−10.2/−4.6		−7.7/−3.2
			LDL (CFB in mmol/l)^c^	—	−0.20		−0.15
SURMOUNT‐J [97] (*n* = 267)	Japanese adults With BMI ≥ 27 kg/m^2^ and ≥ 2 comorbidities^d^ or BMI ≥ 35 kg/m^2^ and at least one comorbidity^d^ mean BMI 33.5 kg/m^2^	72 weeks	Primary outcomes	Tirzepatide 5 mg	Tirzepatide 10 mg	Tirzepatide 15 mg	Placebo
			Superioriority of TZP in body weight CFB (in %) vs. placebo (ETD vs. placebo)	—	−15.8 (−14.0) **	−20.8 (−19.0) **	−1.8
			Superiority of TZP in participants (in %) achieving ≥ 5% weight loss vs. placebo	—	86 OR 27.4 ** (95% CI 9.8, 76.3)	92 OR 51.3 ** (95% CI 16.3, 161.2)	22
			Outcomes on CV risk factors				
			Waist circumference (CFB in cm)	—	−12.7	−16.6	−1.3
			Blood pressure sys/dia (CFB in mmHg)^c^	—	−11.2/−5.9	−12.0/−6.3	1.9/0.5
			Visceral adipose tissue (CFB in %)	—	−39.4	−44.5	−3.4
SURMOUNT‐OSA 1 [91] (*n* = 234)	Adults with obesity (BMI ≥ 30 kg/m^2^) and OSA were not receiving PAP therapy mean BMI 39.1 kg/m^2^	52 weeks	Primary outcome	—	Tirzepatide MTD (10 or 15 mg)		Placebo
			Superiority of TZP in apnoea‐hypopnea index (CFB in n. events in an hour of sleep) vs. placebo (ETD vs. placebo)	—	−25.3 (−20.0)**		−5.3
			Outcomes on CV risk factors				
			Body weight (CFB in %)	—	−17.7		−1.6
			Blood pressure sys/dia (CFB in mmHg)	—	−9.5/−4.9		−1.8/−2.1
			hsCRP (CFB in mg/l)	—	−1.4		−0.7
			Sleep apnoea‐specific hypoxic burden (CFB in % min/h)	—	−95.2		−25.1
SURMOUNT‐OSA 2 [91] (*n* = 235)	Adults with obesity (BMI ≥ 30 kg/m^2^) and OSA were receiving PAP therapy mean BMI 38.7 kg/m^2^	52 weeks	Primary outcome	—	Tirzepatide MTD (10 or 15 mg)		Placebo
			Superiority of TZP in apnoea‐hypopnea index (CFB in n. events in an hour of sleep) vs. placebo (ETD vs. placebo)	—	−29.3 (−23.8) **		−5.5
			Outcomes on CV risk factors				
			Body weight (CFB in %)	—	−19.6		−2.3
			Blood pressure sys/dia (CFB in mmHg)	—	−7.6/−3.3		−3.9/−2.2
			hsCRP (CFB in mg/l)	—	−1.4		−0.3
			Sleep apnoea‐specific hypoxic burden (CFB in % min/h)	—	−103.0		−41.7

*Note:* Results of statistical analysis are reported only for the primary outcome (**p < 0.001).

Abbreviations: BMI: body mass index, CFB: change from baseline, CV: cardiovascular, dia: diastolic, ETD: estimated treatment difference, hsCRP: high‐sensitivity C‐reactive protein, LDL: low‐density lipoprotein, MTD: maximum tolerated dose, OSA: obstructive sleep apnoea, PAP: pressure airway positive, sys: systolic, TZP: tirzepatide.

^a^ Comorbidities include hypertension (treated or ≥ 130/80 mmHg), dyslipidemia (treated or LDL ≥ 4.1 mmol/L or trigl ≥ 1.7 mmol/L or HDL < 1 mmol/L for men, or HDL < 1.3 mmol/L for women), obstructive sleep apnoea or cardiovascular disease (e.g., ischemic cardiovascular disease, New York Heart Association Functional Classification Class I‐III heart failure).

^b^ HbA1c 53–86 mmol/mol (7%–10%), on stable therapy, either diet and exercise alone or oral antihyperglycaemic medication.

^c^ Efficacy estimand.

^d^ Comorbidities include impaired glucose tolerance (defined as having an oral glucose tolerance test 0‐h glucose of at least 6.1 mmol/L or 2‐h glucose of at least 7.8 mmol/L, or both, inclusive of borderline type impaired fasting serum glucose as defined by Japanese clinical practice guidelines for diabetes), hyperlipidaemia (defined as fasting triglycerides of 1.69 mmol/L or greater) and Metabolic dysfunction‐associated steatotic liver disease (defined as having a hepatic fat fraction of 5% or greater as measured by MRI‐proton density fat fraction).

In SURMOUNT‐1, involving adults with obesity (BMI ≥ 30 kg/m^2^) or overweight (BMI ≥ 27 kg/m^2^) and weight‐related complications but without T2D, tirzepatide achieved mean weight reductions up to 20.9% at 15 mg compared with 3.1% with placebo [92]. Importantly, weight loss was also accompanied by reductions in blood pressure (BP), waist circumference, fasting glucose and LDL cholesterol, with increases in HDL cholesterol [92]. Post hoc analyses indicated that this broad improvement in CV risk profile translated into a potential reduction in predicted 10‐year atherosclerotic CV disease (ASCVD) risk, particularly among individuals at higher baseline risk [99].

Subsequent trials confirmed the consistency of these effects across different clinical settings. SURMOUNT‐2 showed substantial, though attenuated, weight loss in obesity and T2D [93], while SURMOUNT‐3 and SURMOUNT‐4 demonstrated that tirzepatide augments lifestyle‐induced weight loss and that continued treatment is necessary to prevent weight regain [94, 95]. Across trials, improvements in BP, lipid profile and T2D prevention (except in SURMOUNT‐2) underscore tirzepatide's substantial CV benefits. Safety remained consistent with other GLP‐1‐based medications, with mainly transient and dose‐dependent GI AEs (nausea, diarrhoea, constipation).

SURMOUNT‐5 directly compared tirzepatide (10 or 15 mg QW) directly with semaglutide (1.7 or 2.4 mg QW) and confirmed superior efficacy, with mean weight loss of −20.2% versus −13.7%, alongside larger reductions in systolic BP and TAGs levels [96]. This translated into a greater estimated 10‐year CV risk reduction (−2.4% vs. −1.4%) using sex‐specific BMI‐based equations [100]. Whether these improvements in surrogate outcomes will translate into reduced hard CV outcomes will be addressed by the ongoing SURMOUNT‐MMO (Morbidity and Mortality in Obesity) trial in individuals with obesity and established or high CV risk without T2D [101].

In SURMOUNT‐OSA 1 and 2, tirzepatide significantly reduced AHI together with body weight, high sensitivity C‐reactive protein (hsCRP), systolic BP and improvements in patient‐reported sleep outcomes. Given the established contribution of intermittent hypoxia and sleep fragmentation to hypertension, arrhythmias, endothelial dysfunction and vascular inflammation [98], these findings suggest that tirzepatide may favourably modify key mechanisms linking obesity, OSA and CV disease. These results led to Food and Drug Administration approval for obesity‐associated OSA, representing the first pharmacological therapy targeting both excess adiposity and OSA severity [102].

## 8 Tirzepatide for the Treatment of T2D

The SURPASS phase 3 program established tirzepatide as a highly effective therapy for T2D, with HbA1c reduction as the primary endpoint across studies, except SURPASS‐J‐COMBO, which primarily assessed safety and tolerability [103]. An overview of the study outcomes is presented in Table 2 [15, 103, 104, 105, 106, 107, 108, 109, 110, 111, 112, 113].

**TABLE 2:** Overview of the SURPASS programme.

Trial	Participant characteristics	Duration	Outcomes	Results in treatment arms (expressed as treatment efficacy estimand)			
SURPASS‐1 [104] (*n* = 478)	Adults with T2D, drug‐naïve mean HbA1c 63.3 mmol/mol mean BMI 31.9 kg/m^2^	40 weeks	Primary outcome	Tirzepatide 5 mg	Tirzepatide 10 mg	Tirzepatide 15 mg	Placebo
			Superiority of TZP in HbA1c CFB (mmol/mol) vs. placebo (ETD vs. placebo)	−20.40 (−20.80) **	20.70 (−21.10) **	22.70 (−23.10) **	0.40
			Change on CV risk factors				
			Body weight (CFB in kg)	−7.0	−7.8	−9.5	−0.7
			Blood pressure sys/dia (CFB in mmHg)	−4.7/−2.9	−5.2/−3.1	−4.7/−3.4	−2.0/−1.4
			LDL (CFB in %)	−6.66	−7.58	−12.43	−1.64
SURPASS‐2 [105] (*n* = 1879)	Adults with T2D on metformin mean HbA1c 67.0 mmol/mol mean BMI 34.2 kg/m^2^	40 weeks	Primary outcome	Tirzepatide 5 mg	Tirzepatide 10 mg	Tirzepatide 15 mg	Semaglutide 1 mg
			Non‐inferiority of TZP 10 or 15 mg (or both) in HbA1c CFB (mmol/mol) vs. semaglutide (ETD vs. semaglutide in mmol/mol)	−22.8 (−2.5) * ^†^	−25.9 (−5.6) ** ^†^	−26.9 (−6.6) ** ^†^	−20.3
			Change on CV risk factors				
			Body weight (CFB in kg)	−7.8	−10.3	−12.4	−6.2
			Blood pressure sys/dia (CFB in mmHg)	−4.8/−1.9	−5.3/−2.5	−6.5/−2.9	−3.6/−1.0
			LDL (CFB in %)	−7.68	−5.59	−5.15	−6.39
			eGFR (CFB in ml/min/1.73 m^2^)	−4.6	−4.8	−5.0	−4.5
			UACR (CFB in %)	−8.5	−1.4	−12.8	−4.3
SURPASS‐3 [106] (*n* = 1444)	Adults withT2D on metformin ± SGLT2i mean HbA1c 65.8 mmol/mol Mean BMI 33.5 kg/m^2^	52 weeks	Primary outcome	Tirzepatide 5 mg	Tirzepatide 10 mg	Tirzepatide 15 mg	Insulin degludec
			Non‐inferiority of TZP 10 or 15 mg (or both) in HbA1c CFB (mmol/mol) vs. insulin degludec (ETD vs. insulin degludec)	−21.1 (−6.4) **	−24.0 (−9.4) **	−26.0 (−11.3) **	−14.6
			Change on CV risk factors				
			Body weight (CFB in kg)	−7.5	−10.7	−12.9	2.3
			Blood pressure sys/dia (CFB in mmHg)	−4.9/−2.0	−6.6/−2.5	−5.5/−1.9	0.5/0.4
			LDL (CFB in %)	−6.01	−5.70	−6.55	−2.71
			eGFR (CFB in ml/min/1.73 m^2^)	−5.9	−4.6	−5.2	−5.6
			UACR (CFB in %)	−9.7	−7.3	−15.8	3.9
SURPASS‐4 [107] (*n* = 2002)	Adults with T2D on metformin and/or SU, and/or SGLT‐2i with high CV risk^a^ mean HbA1c 69.7 mmol/mol mean BMI 32.6 kg/m^2^	52 weeks	Primary outcome	Tirzepatide 5 mg	Tirzepatide 10 mg	Tirzepatide 15 mg	Insulin glargine
			Non‐inferiority of TZP 10 or 15 mg (or both) in HbA1c CFB (mmol/mol) vs. insulin glargine (ETD vs. insulin glargine)	−23.0 (−13.6) **	−26.2 (−16.8) **	−25.6 (−16.1) **	−9.5
			Change on CV risk factors				
			Body weight (CFB in kg)	−7.1	−9.5	−11.7	1.9
			Blood pressure sys/dia (CFB in mmHg)	−2.8/−1.0	−3.7/−0.8	−4.8/−1.0	1.3/0.7
			LDL (CFB in %)	−6.8	−8.3	−7.9	1.4
SURPASS‐5 [108] (*n* = 475)	Adults with T2D on insulin glargine ± metformin mean HbA1c 67.4 mmol/mol mean BMI 33.4 kg/m^2^	40 weeks	Primary outcome	Tirzepatide 5 mg	Tirzepatide 10 mg	Tirzepatide 15 mg	Placebo
			Superiority of TZP 10 or 15 mg (or both) HbA1c CFB (mmol/mol) vs. placebo (ETD vs. placebo)	−24.4 (−14.2) **	−28.3 (−18.1) **	−28.3 (−18.1) **	−10.2
			Change on CV risk factors				
			Body weight (CFB in kg)	−5.4	−7.5	−8.8	1.6
			Blood pressure sys/dia (CFB in mmHg)	−6.1/−2.0	−8.3/−3.3	−12.6/−4.5	−1.7/−2.1
			LDL (CFB in %)	−8.9	−12.8	−15.5	2.8
SURPASS‐6 [109] (*n* = 1428)	Adults with T2D inadequately controlled on basal insulin ± metformin/SU/DPP4 mean HbA1c 72.7 mmol/mol mean BMI 33.2 kg/m^2^	52 weeks	Primary outcome	Pooled tirzepatide (5, 10 and 15 mg)			Insulin lispro
			Non‐inferiority of TZP (pooled cohort) in HbA1c CFB (mmol/mol) vs. insulin lispro (ETD vs. insulin lispro)	−24.7 (−10.7) **			−12.7
			Change on CV risk factors				
			Body weight (CFB in kg)^b^	−6.7	−9.2	−11.0	3.2
			Blood pressure sys/dia (CFB in mmHg)	−7.4 /−2.3	−9.0 /−3.3	−5.9 /−1.0	−0.4/−0.4
			LDL (CFB in %)	−3.9	−2.5	−3.4	4.9
			eGFR (CFB in ml/min/1.73 m^2^)	−3.6	−2.8	−1.9	−2.7
			UACR (CFB in %)	−38.1	−35.4	−30.8	−7.4
SURPASS‐J‐MONO [110] (*n* = 636)	Adults with T2D drug‐naïve mean HbA1c 65.9 mmol/mol mean BMI 28.1 kg/m^2^	52 weeks	Primary outcome	Tirzepatide 5 mg	Tirzepatide 10 mg	Tirzepatide 15 mg	Dulaglutide 0.75 mg
			Superiority of TZP in HbA1c CFB (mmol/mol) vs. dulaglutide (ETD vs. dulaglutide)	−26.0 (11.9) **	−27.9 (−13.8) **	−30.8 (−16.7) **	−14.1
			Change on CV risk factors				
			Body weight (CFB in kg)	−5.8	−8.5	−10.7	−0.5
			Blood pressure sys/dia (CFB in mmHg)	−6.5/−3.2	−8.8/−4.0	−11.0/−5.6	−1.4/0.1
			LDL (CFB in %)	−12.0	−12.8	−19.3	−4.8
SURPASS‐J‐COMBO [104] (*n* = 443)	Adults with T2D on oral agents^c^ Mean HbA1c 70.0 mmol/mol mean BMI 27.9 kg/m^2^	52 weeks	Primary outcome	Pooled all treatment groups (tirzepatide 5, 10 or 15 mg)			—
			Safety and tolerability of TZP^d^	398 (90%) participants completed the treatment and 94% completed the study			—
			Change on CV risk factors				
			Body weight (CFB in kg)	−3.8	−7.5	−10.2	—
			Blood pressure sys/dia (CFB in mmHg)	−8.1/−3.4 (pooled 5, 10 and 15 mg)			—
			LDL (CFB in %)	−13.6	−15.1	−18.0	—
SURPASS‐AP‐Combo [111] (*n* = 917)	Adults with T2D on metformin ± SU mean HbA1c 71.4 mmol/mol mean BMI 27.9 kg/m^2^	40 weeks	Primary outcome	Tirzepatide 5 mg	Tirzepatide 10 mg	Tirzepatide 15 mg	Insulin glargine
			Non‐inferiority in HbA1c CFB (mmol/mol) vs. insulin glargine (ETD vs. insulin glargine)	−24.5 (−14.1) **	−26.7 (−16.3) **	−27.2 (16.8) **	−10.4
			Change on CV risk factors				
			Blood pressure sys/dia (CFB in mmHg)	−6.7/−4.0	−7.2/−3.6	−7.3/−3.4	1.1/0.9
SURPASS‐CN‐INS [112] (*n* = 257)	Adults with T2D inadequately controlled on insulin glargine ± metformin ± SGLT2i mean HbA1c 71.9 mmol/mol mean BMI 27.9 m^2^	40 weeks	Primary outcome	Tirzepatide 5 mg	Tirzepatide 10 mg	Tirzepatide 15 mg	Placebo
			Superiority of TZP 10 or 15 mg (or both) HbA1c CFB (mmol/mol) vs. placebo (ETD vs. placebo)	−23.0 (−13.0) **	−26.1 (−16.1) **	−25.9 (−15.9) **	−10.0
			Change on CV risk factors				
			Body weight (CFB in kg)	−2.7	−4.8	−4.3	1.7
			Blood pressure sys/dia (CFB in mmHg)	−0.48/−1.96	−5.11/−2.88	−1.74/−2.54	1.97/−10.1
			LDL (CFB in %)	−4.5	−0.2	2.2	12.4
SURPASS‐PEDS [113] (*n* = 99)	Young individuals (10–18 years) with T2D inadequately controlled on metformin ± basal insulin mean HbA1c 64.4 mmol/mol mean BMI 35.4 m^2^	52 weeks (30 double‐blind + 22 open‐label)	Primary outcome	Tirzepatide 5 mg	Tirzepatide 10 mg	Tirzepatide 15 mg	Placebo
			Superiority of pooled TZP 5 and 10 mg HbA1c CFB (mmol/mol) vs. placebo at 30 weeks (ETD vs. placebo)	−24.2 (−24.7) **	−25.7 (−26.2) **	—	0.5
			Change on CV risk factors			—	
			Body weight at 30 weeks (CFB in kg)	−5.6	−10.3	—	0.1
			Blood pressure sys/dia at 30 weeks (CFB in mmHg)	−1.0/−2.0	−7.3/−5.3	—	1.3/0.3
			LDL at 30 weeks (CFB in %)	−5.50	−10.82	—	−1.74
			UACR at 30 weeks (CFB in %)	−36.1	−20.5	—	−40.1
SURPASS‐CVOT [15] (*n* = 13 299)	Adults with T2D with established CV disease^e^ Mean HbA1c 68.2 mmol/mol Mean BMI 32.6 kg/m^2^	208 weeks (median duration)	Primary outcome	Tirzepatide (up to 15 mg)			Dulaglutide 1.5 mg
			Non‐inferiority of TZP in composite of death from CV causes, myocardial infarction, or stroke [*n* of patients with events (%)]	801 (12.2%) HR 0.92 * 95% CI (0.83, 1.01)			862 (13.1%)
			Change on CV risk factors				
			HbA1c CFB at 160 weeks (mmol/mol)	−18.1			−9.6
			Weight reduction at 160 weeks (CFB in %)	−11.6			−4.8
			Blood pressure sys at 160 weeks (CFB in mmHg)	−6.2			−4.1
			LDL at 104 weeks (CFB in %)	−1.6			−2.9
			eGFR at 160 weeks (CFB in ml/min/1.73 m^2^)	−5.72			−8.90

*Note:* Results of statistical analysis are reported only for the primary outcome (*p < 0.05, **p < 0.001, †analysis for superiority).

Abbreviations: BMI: body mass index, CFB: change from baseline, CI: confidence interval, CV: cardiovascular, dia: diastolic, DPP4: dipeptidyl peptidase‐4 inhibitor, eGFR: estimated glomerular filtration rate, ETD: estimated treatment difference, HR: hazard ratio, LDL: low‐density lipoprotein, SGLT2i: sodium‐glucose co‐transporter‐2 inhibitor, SU: sulfonylurea, sys: systolic, T2D: type 2 diabetes, TZP: tirzepatide, UACR: urine albumine creatinine ratio.

^a^ Elevated risk of cardiovascular events defined as known coronary, peripheral arterial or cerebrovascular disease or aged 50 years or older with either history of chronic kidney disease and an estimated glomerular filtration rate (eGFR) of less than 60 mL/min per 1.73 m^2^ or history of congestive heart failure (New York Heart Association Class II or III).

^b^ Treatment regimen estimand.

^c^ Oral antihyperglycaemic monotherapy of SU, biguanides, alfa‐glucosidase inhibitors, thiazolidinedione, glinides, or SGLT2 inhibitors.

^d^ As incidence of treatment‐emergent adverse events in the modified intention‐to‐treat (mITT) population.

^e^ CV disease defined as coronary artery disease or myocardial infarction or coronary revascularization or stroke or peripheral artery disease or previous heart failure.

In SURPASS‐1, among drug‐naïve individuals inadequately controlled with diet and exercise, tirzepatide reduced HbA1c by up to 23 mmol/mol at 15 mg, with weight loss ranging from 7.0 to 9.5 kg and no clinically significant (< 54 mg/dL [< 3 mmol/L]) or severe hypoglycaemia [104]. Comparative studies demonstrated superiority of tirzepatide in HbA1c and weight reductions compared with semaglutide 1.0 mg (SURPASS 2) and insulin degludec (SURPASS 3) [105, 106]. Moreover, a continuous glucose monitoring (CGM) sub‐study of SURPASS‐3 confirmed superior CGM‐derived glycaemic control with tirzepatide compared with insulin degludec [114].

These advantages were maintained in more complex settings. In SURPASS‐4, including participants at high CV risk, tirzepatide achieved superior glycaemic control and weight reduction compared with insulin glargine [107]. SURPASS‐5 and SURPASS‐6 demonstrated benefits of tirzepatide in insulin‐treated populations [108, 109]. In particular, SURPASS‐6 showed that tirzepatide improved HbA1c, promoted weight and reduced the risk of hypoglycaemia compared with intensified insulin strategies.

Across SURPASS studies, improvements in waist circumference, BP and lipid profile indicate that tirzepatide's metabolic benefits extend beyond glucose lowering. A pre‐specified meta‐analysis of SURPASS‐1 to −5 showed no increase in MACE with tirzepatide and suggested a trend toward fewer events versus comparators [115]. Notably, post hoc analysis of SURPASS‐4 also identified slower estimated glomerular filtration rate (eGFR) decline and smaller increase in urinary albumin‐to‐creatinine ratio [116]. These benefits appeared consistent across baseline kidney function levels and were likely mediated by improvements in glycaemic control, body weight and BP, though direct renal mechanisms may also contribute.

Finally, the SURPASS‐CVOT has provided more precise evidence regarding the impact of tirzepatide on CV outcomes in individuals with T2D and CV disease, as discussed in the subsequent paragraph.

## 9 Tirzepatide for the Treatment of CV Disease in T2D and the Evolution of CVOTs in T2D

The SURPASS‐CVOT trial represents an important methodological shift in CV outcome assessment in T2D, being the first CVOT in T2D to adopt an active comparator with established CV benefit rather than placebo [15]. Tirzepatide was compared head‐to‐head with dulaglutide, a long‐acting GLP‐1 RA shown to reduce CV events in the REWIND trial [6].

SURPASS‐CVOT enrolled 13 299 individuals with established ASCVD receiving contemporary standard‐of‐care therapy, including substantial sodium‐glucose cotransporter‐2 inhibitors (SGLT2is) exposure (30%) and widespread statin use (86%). The enrolled population reflected long‐standing T2D, with a mean age of 64 years, diabetes duration of 14.7 years, baseline HbA1c of 68 mmol/mol and BMI of 32.6 kg/m^2^.

Tirzepatide met the prespecified non‐inferiority criterion for 3‐point MACE (cardiovascular death, non‐fatal myocardial infarction [MI], or non‐fatal stroke) versus dulaglutide, with a hazard ratio (HR) of 0.92 (95.3% CI 0.83–1.01). Consistent results were observed across individual MACE components, with no statistically significant superiority over dulaglutide. Conversely, all‐cause mortality was reduced by 16% (HR, 0.84; 95.0% CI, 0.75 to 0.94) [15], although this finding should be considered exploratory and warrants further confirmation. Moreover, according to early results announced in July 2025 [117], a pre‐specified indirect comparison of matched patient‐level data from REWIND and SURPASS‐CVOT studies found that tirzepatide reduced the risk of 3‐point MACE by 28% (HR, 0.72; 95.0% CI, 0.55 to 0.94) and all‐cause mortality by 39% (HR, 0.61; 95.0% CI: 0.45 to 0.82) versus a putative placebo. Nevertheless, the use of a putative placebo has several limitations, and these estimates should be interpreted with great caution. Furthermore, a clinically relevant signal emerged in participants with high‐risk CKD, where tirzepatide slowed eGFR decline by 3.54 mL/min/1.73 m^2^ over 36 months versus dulaglutide and reduced the primary composite kidney outcome (HR 0.79 [95% CI, 0.65 to 0.94]) [117]. Whether this renal benefit reflects direct drug effects or is largely mediated by improvements in glycaemic control and body weight remains uncertain and requires dedicated investigation.

Compared with earlier GLP‐1 RAs CVOTs, SURPASS‐CVOT reflects a more contemporary trial paradigm. Most previous CVOTs involving GLP‐1 RAs in T2D were conducted before SGLT2i were widely available and implemented in clinical practice for their cardiorenal benefits and thus represent earlier therapeutic contexts with limited cardioprotective background therapy. Furthermore, the active‐comparator design provides a more rigorous and clinically informative framework. In the context of the well‐established CV benefit of other GLP‐1 RAs, randomization to placebo may be considered unethical, and direct comparison of GLP‐1‐based compounds appears more relevant to clinical decision‐making. Nevertheless, the trial formally established non‐inferiority rather than superiority, and any estimate of placebo‐equivalent benefit remains necessarily indirect.

## 10 Additional Evidence for CV and Renal Protection With Tirzepatide

Beyond T2D‐specific CVOTs, tirzepatide has also been evaluated in obesity‐related HFpEF through the placebo‐controlled phase 3 SUMMIT trial, in which approximately half of participants also had T2D [14].

Over a median follow‐up of nearly 2 years, tirzepatide reduced the composite endpoint of CV death or worsening HF events by 38% versus placebo (HR 0.62; 95% CI 0.41–0.95). The observed benefit was primarily driven by fewer HF hospitalizations (8.0% vs. 14.2%; HR 0.54; 95% CI 0.34–0.85), together with parallel improvements in symptoms and functional capacity, alongside favourable modulation of systemic congestion and inflammation markers, including estimated blood volume, systolic BP and hsCRP [14]. Nevertheless, although based on few events, the number of CV death was numerically higher in the tirzepatide arm (HR 1.58, 95% CI 0.52–4.83), a finding partly influenced by the inclusion of deaths of undetermined cause, unlike the endpoint adjudication adopted in several other HFpEF trials.

SUMMIT also provided indirect insights regarding individuals with reduced kidney function (eGFR < 60 mL/min), showing relative risk reductions for the primary endpoint consistent with those observed in participants with preserved eGFR. However, limited background use of SGLT2i warrants careful interpretation. While evidence for reduced HF hospitalizations is substantial, it simultaneously leaves unresolved whether tirzepatide provides additive benefit when combined with SGLT2i, a cornerstone of evidence‐based HF management.

Overall, SUMMIT supports a potential role for tirzepatide in managing HFpEF, particularly by reducing HF hospitalizations, expanding the cardioprotective paradigm of incretin‐based therapies beyond atherosclerotic disease.

## 11 Beyond Agonism: The Potential of GIPR Antagonism

Despite tirzepatide's metabolic benefits, the precise contribution of GIPR agonism remains unclear. As discussed above, one proposed explanation is that sustained GIPR stimulation may induce receptor desensitization, thereby attenuating the “pro‐obesogenic” signalling of GIP [68]. Supporting this hypothesis, genome‐wide association studies identified reduced‐function variants at the human GIPR locus associated with lower BMI [118, 119].

This has stimulated growing interest in GIPR antagonism as an alternative strategy. Preclinical studies demonstrated that global or adipocyte‐specific deletion of GIPR, or GIP itself, protects against obesity [120, 121]. Monoclonal antibodies (mAbs) targeting GIP or GIPR mitigate weight gain and improve metabolic parameters in mice, with GIPR‐specific Abs also modestly reducing food intake [122, 123]. Notably, combining GIPR antagonism with GLP‐1R agonism potentiates and sustains these effects [41].

Although some evidence suggests that GIPR agonism and antagonism may influence body weight via similar mechanisms [78], a recent murine study found that they act through distinct neuronal pathways, with GIPR antagonism—but not agonism—requiring GLP‐1R signalling to modulate energy metabolism [124].

Potential relevance also extends to the CV system, where inhibition of GIP signalling improved survival and limited adverse ventricular remodelling following experimental MI [51].

In this context, maridebart cafraglutide (MariTide, or AMG‐133), comprising two identical GLP‐1 analogues conjugated to a GIPR antagonist mAb, has emerged as a promising therapeutic candidate. Phase 1 data showed an acceptable safety profile, with dose‐dependent weight reduction up to −14.5% after 12 weeks, sustained for up to 150 days after treatment discontinuation. Its long half‐life (~21 days) also supports monthly or less frequent dosing [125].

Phase 2 findings extended this signal into the clinical setting. In a 52‐week trial involving 592 adults with overweight or obesity, including 127 with T2D, MariTide was administered at varying doses and schedules (140–420 mg every 4 or 8 weeks, with or without escalation) and compared with placebo [126]. Based on the treatment policy estimand (intention‐to‐treat analysis), weight reductions in the obesity cohort ranged from −12.3% (95% CI, −15.0 to −9.7) to −16.2% (95% CI, −18.9 to −13.5) with MariTide, versus −2.5% (95% CI, −4.2 to −0.7) with placebo. In the obesity–diabetes cohort, weight reductions ranged from −8.4% (95% CI, −11.0 to −5.7) to −12.3% (95% CI, −15.3 to −9.2) with MariTide versus −1.7% (95% CI, −2.9 to −0.6) with placebo, accompanied by HbA1c reductions ranging from −13.1 to −17.5 mmol/mol. Additional improvements in waist circumference, BP, CPR and lipid profile reinforce the possibility that GIPR antagonism may favourably influence multiple determinants of cardiometabolic risk. Better tolerability was observed with lower starting doses and gradual dose escalation [126].

The ongoing phase 3 programme is assessing MariTide's efficacy and safety as a treatment for obesity, including the MARITIME‐CV trial, which aims to demonstrate superiority versus placebo for both 3‐point and 5‐point MACE endpoints (all‐cause death, MI, ischemic stroke, coronary revascularization, or HF).

## 12 Conclusions and Future Perspectives

The mechanisms linking GIPR agonism to CV and renal protection remain incompletely understood. Available preclinical and clinical studies suggest pleiotropic effects encompassing anti‐inflammatory, endothelial and metabolic pathways, although the relative contribution of direct receptor‐mediated actions versus secondary effects related to weight loss and glycaemic improvements remains difficult to define. While adding GIPR agonism to GLP‐1R agonism improves multiple cardiometabolic risk factors, it has not demonstrated superiority in CV outcomes in T2D. Ongoing outcome trials will therefore be critical: SURMOUNT‐MMO is expected to clarify whether tirzepatide confers CV protection in obesity, while the TREASURE‐CKD will directly assess its impact on renal outcomes [127].

Another major unresolved issue concerns the interaction between tirzepatide and SGLT2i. Given their complementary mechanisms, combined therapy may theoretically provide broader cardiorenal protection than either class alone. However, robust prospective evidence remains limited, and future studies will need to establish whether additive or synergistic benefit truly exists.

Beyond tirzepatide, a broad and compelling pipeline, targeting GIPR through both monoagonism and multi‐agonism, is currently under development [128]. A logical advance beyond GIPR/GLP‐1R agonism is the development of retatrutide—a single agent targeting GLP‐1R, GIPR and GcgR—leveraging GcgR‐mediated increases in energy expenditure and reduced food intake [129]. Retatrutide is being evaluated in the phase 3 TRIUMPH programme [130], including CV and renal outcome trials, following phase 2 findings demonstrating superior weight loss and improvements in cardiometabolic parameters versus tirzepatide [131].

At the same time, one of the most conceptually intriguing unresolved questions remains why both GIPR agonism and antagonism, when combined with GLP‐1R agonism, appear capable of producing similarly substantial weight reduction. The clinical translation of GIPR antagonism through phase 1 and phase 2 MariTide has intensified this apparent paradox. In light of the debated role of GIP in the CV system and of the non‐inferiority of tirzepatide in SURPASS‐CVOT, it remains particularly relevant to determine whether inhibition of GIP signalling could provide CV benefit beyond that already established for GLP‐1 RAs.

Special consideration is also warranted in older adults, in whom obesity frequently coexists with frailty and sarcopenia, complicating therapeutic management [132]. In this setting, the cardioprotective effect of GLP‐1 RAs appears independent of weight loss, suggesting CV benefits at smaller doses and less weight reduction [133].

Expanding the therapeutic landscape, individuals with T1D represent a distinct population characterized by a rising prevalence of obesity and insulin resistance [134]. Incretin‐based therapies may provide a valuable adjunctive strategy by improving metabolic parameters associated with cardiorenal risk, but dedicated clinical trials will be required to establish their efficacy and safety in this population and to determine their potential protective effects on CV and renal outcomes.

In conclusion, adding GIPR agonism to GLP‐1R agonism clearly improves cardiometabolic risk factors, yet its specific contribution to CV protection remains uncertain. At the same time, promising findings on GIPR antagonism open intriguing avenues for further investigation, within an apparently paradoxical context in which both GIPR agonism and antagonism may be therapeutically relevant. Ongoing and future research is warranted to elucidate mechanisms, assess potential additive or synergistic benefits with established therapies, and evaluate long‐term efficacy and safety of GIPR‐targeted agents across different populations.

## Author Contributions

M.D.F. contributed to the design of the review and drafted the initial version of the manuscript. All authors critically revised the manuscript and approved the final version for submission.

## Funding

The authors have nothing to report.

## Conflicts of Interest

M.d.F. has received honoraria for lecturing and travel grants from AstraZeneca, Eli Lilly, Novo Nordisk, Sanofi; L.L.H. has no conflict of interest; C.R.S. reports personal fees and/or institutional research support from Novo Nordisk and Eli Lilly, outside the submitted work; F.P. receives clinical trial funding, advisory board and lecture fees from Abbott, AstraZeneca, Eli Lilly, Novo Nordisk, Roche, Sanofi; A.A. has served on an event adjudication committee for Amylyx Pharmaceuticals and as consultant to, received lecture fees from and served on advisory boards for Eli Lilly and Novo Nordisk.

## Data Availability

Data sharing not applicable to this article as no datasets were generated or analysed during the current study.
